# Looking at COVID-19 from a Systems Biology Perspective

**DOI:** 10.3390/biom12020188

**Published:** 2022-01-22

**Authors:** Emily Samuela Turilli, Marta Lualdi, Mauro Fasano

**Affiliations:** Department of Science and High Technology, University of Insubria, I-21052 Busto Arsizio, Italy; esturilli@gmail.com (E.S.T.); marta.lualdi@uninsubria.it (M.L.)

**Keywords:** COVID-19, systems biology, proteomics, interactomics, drug repurposing, network medicine, disease classifiers

## Abstract

The sudden outbreak and worldwide spread of the SARS-CoV-2 pandemic pushed the scientific community to find fast solutions to cope with the health emergency. COVID-19 complexity, in terms of clinical outcomes, severity, and response to therapy suggested the use of multifactorial strategies, characteristic of the network medicine, to approach the study of the pathobiology. Proteomics and interactomics especially allow to generate datasets that, reduced and represented in the forms of networks, can be analyzed with the tools of systems biology to unveil specific pathways central to virus–human host interaction. Moreover, artificial intelligence tools can be implemented for the identification of druggable targets and drug repurposing. In this review article, we provide an overview of the results obtained so far, from a systems biology perspective, in the understanding of COVID-19 pathobiology and virus–host interactions, and in the development of disease classifiers and tools for drug repurposing.

## 1. Introduction

Coronaviruses (CoVs) are a large family of viruses, causing mild–severe respiratory tract infections in mammals [1]. In the past two decades, highly pathogenic CoVs emerged from animal reservoirs, such as bats or civets, and caused severe respiratory syndromes [2]. Severe acute respiratory syndrome (SARS) and Middle East respiratory syndrome (MERS) were examples of emerging zoonotic CoV infections capable of person-to-person transmission, which resulted in substantial effects on patients’ health and socioeconomic factors [3]. Although other human pathogenic coronaviruses cause very mild symptoms, patients with SARS-CoV or MERS-CoV infections developed severe acute respiratory disease with multi-organ failure. The case fatality rates of SARS and MERS were approximately 10% and 35%, respectively [4,5]. By the end of 2019, Chinese health authorities informed the World Health Organization (WHO) about a new, severe viral pneumonia associated with a novel CoV [6]. This CoV showed nucleotide sequence similarity greater than 80% with SARS-CoV [7]. Nevertheless, the new SARS-CoV-2 appeared soon to be more contagious, with a worldwide spread of 315.3 million confirmed cases and 5.5 million deaths as of 14 January 2022 (https://covid19.who.int/; accessed on 14 January 2022). Since its first detection (China, December 2019, “Alpha” WHO label), four variants of concern (VOC) emerged within two years (https://www.ecdc.europa.eu/en/covid-19/latest-evidence/diagnostic-testing; accessed on 13 January 2022). For VOC (Table 1), clear evidence exists indicating a significant effect on transmissibility, severity, and/or immunity, which is likely to have an epidemiological impact. One common feature of these variants is the accumulation of mutations in the gene encoding the spike protein, which mediates the interaction between the virus and the host target cells. The appearance of new variants displaying higher transmissibility, causing more severe symptoms, or showing an antigenic profile capable of reducing the sensitivity of diagnostic tests and/or the effectiveness of vaccines, represents the biggest challenge of the present pandemic.

The diagnosis of SARS-CoV-2 infection is currently obtained by several different techniques (Table 2), even though nucleic acid amplification tests (NAATs) are the reference standard (https://www.who.int/publications/i/item/WHO-2019-nCoV-lab-testing-2021.1-eng; accessed on 13 January 2022). Indeed, the amplification and detection of viral RNA in specimens guarantees the highest diagnostic sensitivity, also in the presence of newly emerging variants. Optimal specimens for the detection of SARS-CoV-2 are collected from the upper respiratory tract (e.g., nasopharyngeal swab, oropharyngeal swab, nasopharyngeal aspirate, nasal wash). Saliva was also suggested as a specimen when testing for SARS-CoV-2 [23,24]; however, only assays detecting SARS-CoV-2 RNA should be used for this specimen type, as the sensitivity of antigenic tests is not sufficient.

The disease caused by SARS-CoV-2 (coronavirus disease 2019; COVID-19) immediately appeared to be heterogeneous in terms of transmission [25], severity [26], and outcome predictability [27]. With rapid antigenic tests being available [24,28,29,30,31], a thorough screening allowed the identification of several subjects being infected with SARS-CoV-2 and showing a very mild form of COVID-19 or no symptoms at all [32,33]. By contrast, hospitalization and intensive care are needed for severe COVID-19 patients, whose symptoms are sometimes difficult to treat with available anti-inflammatory drugs and can ultimately lead to death [34]. Moreover, the risk of morbidity and mortality due to COVID-19 increases dramatically in the presence of some coexisting illnesses, such as hypertension, asthma, diabetes mellitus, cardiovascular or cerebrovascular disease, chronic kidney disease, and malignancy [35].

When a perturbation is applied to a complex, non-linear system, the expected outcome is a highly variable inter-individual response, which reflects different severities of the disease and different responses to treatment [36,37]. This suggests taking advantage of “omics” approaches and systems biology to understand the pathobiology and implement predictive tools for disease subtype classification. Indeed, omics strategies (e.g., transcriptomics, proteomics, metabolomics) can be used for the unbiased analysis of any complex biological samples (nasopharyngeal swabs, saliva, biofluids, tissues) aimed at the identification of pathogenetic mechanisms and/or specific biomarkers for disease severity. As an example, by comparing mild and severe COVID-19 patients, proteins, cytokines, and metabolites, whose levels in biofluids correlate with the presence of severe symptoms, can be identified, thus guiding patient stratification and the choice of the most appropriate pharmacological treatment. Such information cannot be obtained with classical targeted approaches, because the molecular factors involved are not known a priori.

In addition, the worldwide health emergency requires the quick availability of effective therapeutic strategies, which suggests the use of drug repurposing tools to identify candidate novel therapies [38]. 

**Figure 1:**
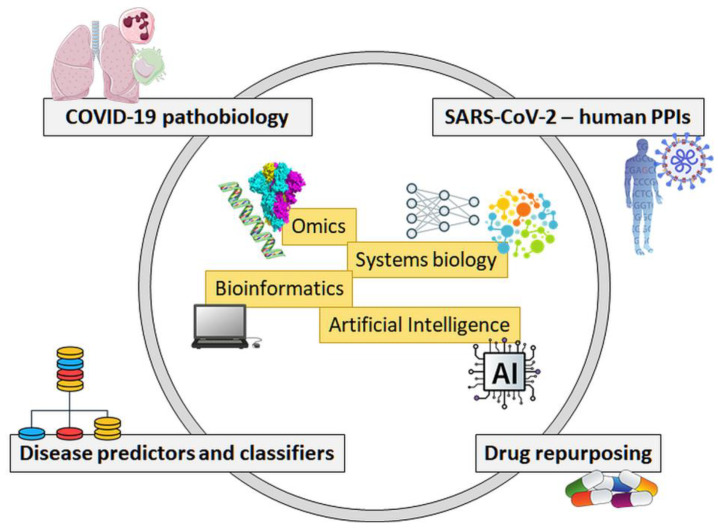
The four main objectives of the battle against SARS-CoV-2 and COVID-19 from a systems biology perspective. PPIs—protein–protein interactions.

As a whole, the effort of omics strategies (especially proteomics and interactomics) and systems biology in the battle against SARS-CoV-2 and COVID-19 is targeted to these main objectives: (i) the comprehension of the pathogenetic mechanisms following SARS-CoV-2 infection; (ii) the understanding of the complex system where the virus meets the host cell, based on protein–protein interactions; (iii) the definition of predictors and/or classifiers for the identification of disease subtypes with distinct outcome; (iv) the implementation of artificial intelligence (AI) procedures for drug repurposing (Figure 1). Here, we provide a review of the results obtained in these four fields so far.

## 2. The Pathobiology of COVID-19 Investigated by Proteomics

The advent of the big data era recently imposed a shift in the scientific thought and approach, switching from the classical hypothesis-driven deductive approach to a data-driven inductive approach. Indeed, “omics” techniques (e.g., proteomics, genomics, transcriptomics, metabolomics) generate huge datasets from which the significant experimental observations are extracted by data reduction and then used to draw inferences on the perturbed mechanisms. Correlation among observations can identify pathways behind them thanks to complex statistical algorithms [39], and meaningful models can be obtained at the network complexity level [40]. Indeed, omics datasets are efficiently represented and analyzed in the form of networks where nodes represent the observations (proteins, genes), and edges represent the associations among them. Notably, the combination of genomics and quantitative approaches to network-based analysis contributed to pushing forward the frontiers of network medicine [41]. The multiplicity of factors that can alter a complex system require, indeed, a multifactorial approach, characteristic of network medicine, to identify functional connections that link the clinical phenotype to such multiple factors [42].

Proteomics and interactomics rely on the mass spectrometry (MS)-based identification and quantification of proteins. The COVID-19 MS coalition was launched aiming at providing molecular level information on SARS-CoV-2 in the human host and revealing pathophysiological and structural information to treat and minimize COVID-19 [43]. This consortium is constituted by about 600 scientists from 60 countries and made all studies publicly available in open-access databases such as the PRIDE repository (http://www.ebi.ac.uk/pride; accessed on 13 January 2022) [44]. Thousands of papers have been published within the last two years reporting the results of proteomics studies performed in different patient groups (mild, moderate, and severe COVID-19), using different experimental models and specimens (from SARS-CoV-2 infected cells to biofluids collected from patients), employing and developing different strategies with different aims (biomarker research, patient stratification, discovery of therapeutic targets).

From clinical observations, it is now clear that severe COVID-19 is associated with an acute phase response, often termed as “cytokine storm”, prothrombotic immunopathology, and lymphopenia, which can culminate in multiple organ dysfunction and, in some cases, death [45]. Moreover, the viral infection can result in lung, heart, and brain damage, which increases the risk of long-term illnesses. Notably, it has also been proposed that SARS-CoV-2 infection may trigger autoimmunity (Guillain-Barré syndrome, systemic lupus erythematosus) through cross-reactivity with host cells [46]. In this context, the main impact of proteomics studies was the possibility to shed light on molecular mechanisms underlying the pathobiology of COVID-19—partially explaining the high heterogeneity in clinical outcomes. Early proteomics studies of the diseased tissue (lung) [47,48] unveiled cathepsin B, cathepsin L, proteins involved in the NF-κB pathway, several interleukins, matrix metalloproteases, and other proteins secreted by neutrophils and macrophages as biomarkers for COVID-19 disease severity. These molecules correlate with the pathogenetic mechanisms of neutrophil extracellular traps (a process that allows for selective elimination of the pathogens minimizing host cell damage), overproduction of proinflammatory cytokines, increased risk of clot formation, platelet activation, and fibrosis associated with severe COVID-19. The proteomics analysis of SARS-CoV-2-infected cells (e.g., Caco-2 cells) unveiled the main molecular pathways driving the infection: protein translation, RNA splicing, glycolysis, and nucleotide synthesis [49,50]. Strikingly, the use of specific inhibitors (i.e., chemicals routinely used for the general inhibition of protein translation, glycolysis, and nucleotide synthesis for research use) at non-toxic concentrations has been proven to prevent viral replication. However, these drugs cannot be used as therapeutics. Eventually, the proteomics of patient-derived biofluids unveiled some biomarkers for patient stratification and disease grade classification. In blood samples, Shu and coworkers demonstrated a significant enrichment in processes involved in inflammation, migration, and degranulation of immune cells, complement system, coagulation cascade, and energy metabolism [51]. Of note, platelet degranulation and the complement and coagulation cascades were the most enriched, with proteins involved in these processes more significantly altered in severe cases versus mild cases.

## 3. Understanding Virus–Host Interaction in a Complex System View

### 3.1. The Network-Based Analysis of Omics Data

The multifactorial approach of network medicine is based on the identification of functional connections that link the clinical phenotype to multiple factors. This aim is achieved by integrating protein–protein interactions (PPIs), expression data, and gene regulatory circuits [41]. Protein networks are usually built starting from a list of selected proteins, which often is the result of a feature selection/extraction procedure applied to omics datasets. Namely, among all proteins identified and quantified by MS (typically one thousand or more), those that are significant to describe the system must be fished out by using a strategy for data reduction [39]. Several methods exist, classified as (i) feature subset selection methods (e.g., filters, wrappers, embedded methods), which select a subgroup of the original dataset by removing non-relevant or redundant proteins, and (ii) feature extraction methods (e.g., discriminant analysis, principal component analysis), which create new variables as combination (aggregation, transformation) of the existing ones. Independently of the method of choice, the result is a reduced dataset of selected proteins that are used to query a suitable database to retrieve protein–protein interaction information; the latter is used to create associations (edges) among the proteins (nodes) within the network. It is worth noting that, depending on the database of choice, the interaction between two proteins does not necessarily mean they are physically binding to each other. Indeed, an edge can represent not only a known physical interaction (experimentally determined, retrieved from curated databases) but also a predicted interaction (gene neighborhood, gene co-occurrence, co-expression, protein homology, text mining). The result is a PPI network that may be further expanded by adding first interactors that might join isolated or distant nodes. The rationale of this approach is that false-positive proteins, i.e., proteins that were identified as phenotype-correlated by chance, are likely excluded from the network, whereas proteins that for several reasons were not detected as phenotype-correlated are now reconnected to the network [36,40]. A functional analysis of the resulting network is eventually performed with the aim of identifying biochemical pathways and processes significantly related to the selected proteins. A standard functional analysis usually consists of an over-representation analysis (ORA) based on the Fisher’s exact test or a gene set enrichment analysis (GSEA) [52]. Briefly, in ORA the hypergeometric Fisher’s test is used to compute a p-value for each overrepresented pathway; this p-value is calculated from the number of proteins in the experimental list (network) and in a reference database, assigned or not to a given pathway. Instead, GSEA works by ranking all proteins in the experimental list according to a parameter (usually the level of differential expression) and tests whether any annotated gene set is ranked unexpectedly high or low through running sum statistic. Independently of the approach chosen, the result of the functional analysis of the network is a list of significantly enriched pathways, which may help in highlighting crucial mechanisms related to the system under study.

### 3.2. A Network of Known Physical Interactions between Human and SARS-CoV-2 Proteins

**Figure 2:**
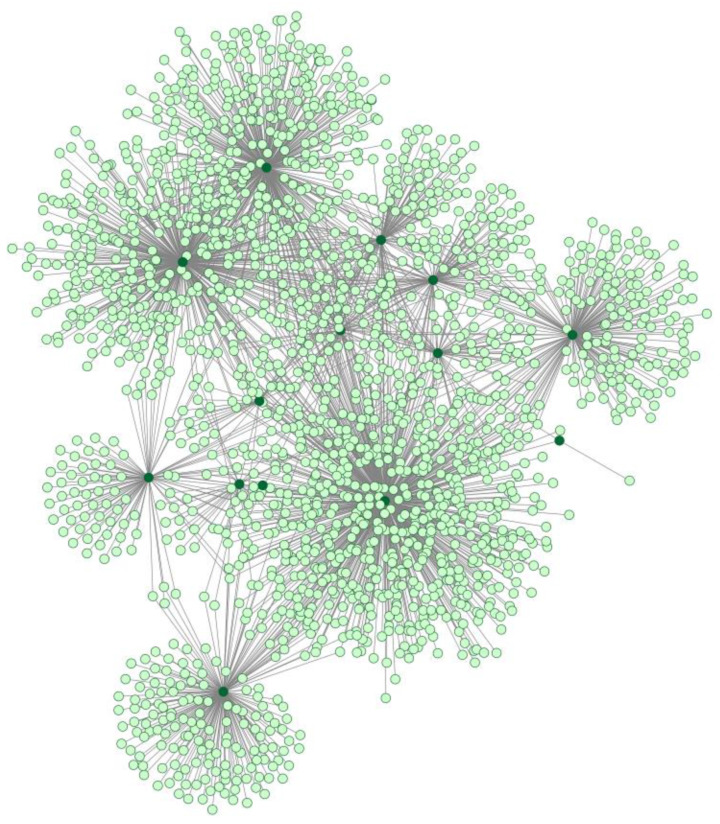
The protein–protein interaction network obtained from IMEx. Dark green nodes represent SARS-CoV-2 proteins, whereas light green nodes are human proteins.

The International Molecular Exchange (IMEx) Consortium provided researchers with a dedicated collection of over 4400 binarized interactions between human proteins and SARS-CoV/SARS-CoV-2 proteins extracted from 151 publications, as of November 2020 [53]. Uniprot dedicated a specialized database for SARS-CoV-2 or COVID-19-related proteins (https://covid-19.uniprot.org/; accessed on 14 January 2022), with 120 annotated entries (89 human, 16 SARS-CoV-2, and 15 SARS-CoV proteins, to date). Based on a query performed on 14 January 2022, 4410 interactions were found in IMEx between the 16 annotated SARS-CoV-2 proteins and human proteins. This search identified 1933 human protein interactors and 2525 non-redundant edges (Figure 2).

Usually, proteins are supposed to interact because they are observed to accordingly change in their levels when the system is perturbed. Instead, one may identify which are the interactors of specific proteins, such as the SARS-CoV-2 spike protein, to get a more dynamic and complete picture of viral protein–host protein interactions. In this frame, interactomics approaches are essential to study the assembly of specific protein complexes and to highlight interaction changes that can discriminate between two conditions or, more specifically, that are involved in the development of diseases [54]. Several approaches have been recently developed to detect PPIs and populate specific interactomes. These approaches can be roughly classified as follows: (i) in silico methods, which consist of text mining and computer simulation; (ii) in vivo methods (e.g., yeast two-hybrid, protein-fragment complementation assay, mammalian protein–protein interaction trap), which can be performed on intact living organisms; (iii) in vitro methods (e.g., tandem affinity purification-mass spectroscopy, protein microarray), which are performed in a controlled environment outside a living system [55]. The resulting interactomes can be then represented as networks and functionally analyzed with the tools of systems biology.

SARS-CoV-2 requires host receptor proteins such as angiotensin-converting enzyme 2 (ACE2) to bind with the spike protein. ACE2 naturally protects against acute lung injury [56], which explains the increased lung pathophysiology and pathobiology (e.g., acute respiratory distress syndrome, pneumonia, and lung injury) due to dysregulation of ACE2 resulting from binding to the spike protein of SARS-CoV-2 [57]. To better understand the regulatory network behind ACE2, Lite and coworkers built a PPI network by retrieving ACE2 first and second interactors and mapped with RNA-Seq lung expression data [58]. Overall, the interactome data revealed that AGT (angiotensinogen), LAMAS (laminin subunit alpha 1), NTS (neurotensin) and GHRL (ghrelin and obestatin prepropeptide) represent direct mediators, which are assumed to be activated upon viral binding of the SARS-CoV-2 with the ACE2 receptor. More recently, Bamberger and coworkers developed a novel proteome-based cell-type set enrichment analysis (pCtSEA), in order to hypothesize how the tropism of the virus might change due to mutations in the spike protein. The rationale of this approach is that the host interactome determines whether an infection is productive or not. With pCtSEA they concluded that the host interactome of the spike protein may extend the tropism of SARS-CoV-2 beyond epithelial airway cells to other cell types, including macrophages and epithelial cells in the nephron [59]. 

Gordon and coworkers built a SARS-CoV-2 PPI map for the identification of druggable human proteins or host factors, relying on affinity-purification mass spectrometry. Out of the 332 PPI identified, 66 components were known to be targeted by 69 antiviral agents, mainly belonging to mRNA translation inhibitors and regulators of sigma-1 and sigma-2 receptors [60]. Previously published protein–protein interaction and gene expression data from other human coronavirus infections have been essential in the first phases of the pandemic to define functionally enriched host–pathogen network models, allowing the prediction of the SARS-CoV-2 interactome. In this context, Perrin-Cocon et al. [61] selected a set of molecules from published in vitro screenings of chemical libraries that were identified for their antiviral activity against at least one coronavirus and relied on the Drug Repurposing Hub database (https://clue.io/repurposing; accessed on 14 January 2022) [62], developed by the Broad Institute to search for their cellular targets. Results overlapping with the previously generated list of host proteins interacting with coronaviruses have been used to identify candidate antiviral drugs for SARS-CoV-2. Furthermore, the network-based model developed by Messina et al. provided an in-depth comparison of the 3D structure of the SARS-CoV-2 S-glycoprotein to the corresponding SARS-CoV, MERS-CoV, and HCoV-229E [63].

## 4. Computational Methods and Omics for COVID-19 Research

### 4.1. Predictors and Classifiers for COVID-19 Severity and Outcome

Systems biology provides a variety of models and methodologies to extract predictive features from holistic datasets, such as those arising from proteomics, metabolomics, or RNA-seq. In particular, “omics” analysis of either affected tissue (lung) or peripheral biofluids and specimens (serum, plasma, or blood cells) may lead to AI tools to predict disease severity and outcome, or to classify subjects in terms of drug response. 

By comparing serum profiles of 28 severe COVID-19 patients, 28 healthy subjects, 25 non-COVID-19 patients with similar symptoms and 25 non-severe COVID-19 patients, Shen and coworkers built a random forest machine learning model to predict disease severity [64]. The approach was based on proteomics and metabolomics data from 18 non-severe and 13 severe patients, leading to the identification of 93 proteins and 204 metabolites showing differential abundance in severe COVID-19 patient sera. Among them, 29 important variables, including 22 proteins and 7 metabolites, were used to build the classifier. This model showed an Area Under the receiver operator characteristic (ROC) Curve (AUC) of 0.957 in the training set and led to two misclassifications in a test cohort of 10 unknown subjects. The significance of the 29 molecules signature was assessed by random resampling. 

The integration by computational methods of clinical measurements, immune cells characterization, and plasma multi-omics of serial blood samples of 139 COVID-19 patients representing all levels of disease severity allowed Su and coworkers to identify a major shift between mild and moderate disease [65]. Specifically, the authors constructed a cross-omics interaction network that revealed an orchestration between increasing COVID-19 severity, elevated inflammation, and loss of key circulating nutrients. Moreover, the plasma multi-omics profiles described a marked similarity between moderate and severe COVID-19, whereas a sharp difference between mild and moderate infections was observed. This major shift consisted in the preferential loss of lipids, amino acids, and xenobiotic metabolism, with concomitant elevation of inflammatory cytokines. With a similar approach, Shu and coworkers obtained plasma proteomics profiles of COVID-19 patients to train a classifier for clinical outcome prediction [51]. A biomarker panel composed of 11 proteins was identified and several protein combinations were tested in the prediction of distinct outcome parameters based on the highest AUC after 5-fold cross-validation. Several differentially abundant host proteins were directly connected to platelet degranulation, complement cascade, and inflammation, as assessed by GSEA and also reported by Messner and coworkers [66]. In this paper, the authors described an ultra-high-throughput proteomics platform to screen serum or plasma of 180 subjects per day. Using this platform, they identified a panel of 27 biomarkers for the classification of severity grade. This panel included complement factors, the coagulation system, modulators of inflammation, and pro-inflammatory factors upstream and downstream of interleukin 6 [66]. Chen and colleagues added extracellular RNA in the integration of proteome, transcriptome, and clinical data, observing different activation of the inflammatory response in mild and severe disease [67]. Significantly changing molecules allowed the definition of a classifier to predict the prognosis of COVID-19. The combination of proteomics and immunology through computational tools was also exploited by Tilocca and coworkers, who described present immunoinformatics approaches and their potential in contrasting COVID-19, in particular through the prediction of B- and T-epitopes [68].

Serum proteomics profiling can also lead to specific disease classifiers able to distinguish between early stage COVID-19 and flu. Hou and coworkers identified a set of 132 differentially expressed proteins dealing with inflammation and immune signaling in 15 patients diagnosed with SARS-CoV-2 compared with a group of 6 patients with FluA, 3 patients with Flu-B and 4 patients with respiratory syncytial virus (RSV). They also found that neutrophils and lymphocytes levels correlated with the CCL2- and CXCL10-mediated cytokine signaling pathways [69]. 

### 4.2. Discovery of Therapeutic Targets from Proteome Profiling of Infected Tissue

Cells respond to viral infection by dysregulating the levels of several host proteins. Therefore, proteome profiling followed by a functional enrichment analysis with systems biology tools may indicate potential therapeutic targets.

It is known that SARS-CoV-2 replicates in intestinal cells [70] and it is frequently detected in feces [71]. Since human colon epithelial carcinoma Caco-2 cells were widely used to investigate host cell response to SARS-CoV infection [72], Bojkova and coworkers investigated the proteome of Caco-2 cells infected with SARS-CoV-2 [73]. The proteome profile of host cells was monitored at different time points after infection. Results indicated several pathways that include druggable targets, with a focus on host cell translation inhibitors. Leng and coworkers, on the other hand, profiled the proteome of fresh lung tissue obtained from deceased patients, showing an extensive dysregulation [48]. Results were interpreted in terms of structural changes in the lung tissue that correlate with disease symptoms, in particular, those involving the extracellular matrix, surfactant proteins that allow gas exchange, and the coagulation pathway. 

In order to define the host immune response implicated in COVID-19 fatality, a 166-gene signature was designed by analyzing over 45,000 transcriptomics datasets of viral pandemics using ACE2 as a seed gene [74]. A subset of 20 genes was proposed as a signature for disease severity. This study led to the identification of neutralizing antibodies or directly acting antiviral agents (e.g., EIDD-2801/Molnupiravir, now approved for the treatment of mild-to-moderate COVID-19 in adults with high risk for progression to severe COVID-19) as signature modifiers.

Very recently, Liu and coworkers exploited a MS-based interactomics approach to identify candidate targets for antiviral therapy among proteins involved in the processing of viral proteins, which is a crucial aspect for productive infection [75]. In order to map the PPIs relevant to viral processing, they applied both affinity purification mass spectrometry and the complementary proximity-based labeling MS method on 29 viral ORFs and 18 host proteins with roles in viral replication. The result was a list of 693 hub proteins sharing interactions with both viral and host baits; functional enrichment analysis highlighted 3 main pathways, namely RNA transportation, endocytosis, and protein processing in the endoplasmic reticulum. This list of candidate targets also served as a resource for rational drug repurposing via a virtual screening approach, by which the authors suggested repurposing of 59 antiviral compounds for 15 protein targets.

## 5. Artificial Intelligence and Network Medicine for a Therapy to Cure COVID-19

### 5.1. Drug Repurposing

To date, 4101 interventional studies related to COVID-19 have been reported on clinicaltrials.gov (14 January 2022). Among the 1720 clinical studies where the intervention is classified as “drug”, 126 involve monoclonal antibodies mostly aimed at targeting SARS-CoV-2 proteins or inflammatory cytokines. The vast majority of studies concerning small-molecule drugs is focused on compounds that were discovered and developed prior to the COVID-19 pandemic, already marketed and approved for at least one clinical indication. Only a few study medications have been designed ad hoc with the primary aim to target COVID-19. Given the impetuous nature of the pandemic, it is not surprising that most drugs under clinical investigation belong to a repurposing program. The journey to bring a new medication from de novo discovery to the marketplace takes at least 10 years and the average cost for the whole R&D process is estimated to be around USD 2.6 billion. Drug repurposing (sometimes also called drug repositioning, re-profiling, or re-tasking) is a time- and cost-effective strategy for discovering new clinical indications for approved (and investigational) drugs that were originally developed for a different medical condition, not necessarily belonging to the same therapeutic area. Approved drugs guarantee already established pre-clinical and clinical safety, toxicological, and pharmacokinetics data, allowing researchers to speed up non-clinical assessments and optimizations (especially when molecular targets are the same) and to avoid drug testing in healthy volunteers (phase I clinical trials). This de-risking strategy is particularly effective when no additional chemistry, manufacturing, and controls (CMC) effort (e.g., a new formulation, different delivery route, and/or release profile) must be taken into account.

It is not uncommon for big pharmaceutical companies to structure alternative positioning plans of their new drug candidates while still in the clinical trials process for the original indication, especially in the oncology space. An example is Merck’s programmed death receptor-1 (PD-1) inhibitor pembrolizumab (sold under the brand name Keytruda^®^), which was originally approved for previously treated unresectable or metastatic melanoma in 2014 and is now approved for the treatment of a total of 18 cancer types, including non-solid tumors, such as Hodgkin lymphoma.

Many blockbuster drugs are the result of a repurposing process, with serendipity being a frequent part of such discoveries, especially in the past century. Amantadine was first approved in the 1960s for flu prophylaxis [76] but is now no longer recommended as an antiviral agent. Indeed, amantadine is now mostly known as an antiparkinsonian agent. In 1968, a 58-year-old woman affected by Parkinson’s Disease (PD) had noticed an improvement in rigidity, tremor, and akinesia while taking amantadine for the treatment of a flu infection and reported that her symptoms worsened upon stopping the medication [77]. Several animal and human studies were performed to shed light on such an effect and led to the FDA approval of amantadine for the treatment of motor symptoms in PD in 1973, which was also followed by the FDA approval of an extended-release formulation (Gocovri^®^) for levodopa-induced dyskinesia in 2017. Further outstanding examples among several successful repurposing stories are certainly the phosphodiesterase-5 (PDE5)-inhibitor, sildenafil (commercially known as Viagra^®^, approved in 1998 for the treatment of erectile dysfunction, but originally designed to treat systemic hypertension and angina), and the well-known immunomodulatory drug thalidomide (Thalomid^®^, approved in 2006 for the treatment of multiple myeloma in combination with dexamethasone, but marketed—and subsequently withdrawn—in the 1950s as a sedative).

However, despite its versatility, the repurposing approach has been often ignored by healthcare investors, mainly due to misperceptions related to intellectual property protection, pricing strategies, and discovery approaches: during 2004–2013, nearly 80% of venture capital funding went toward novel R&D of new chemical entities with no prior regulatory approval, as opposed to improvements of approved drugs (including delivery, repurposing, and reformulation) [78]. In the last few years, thanks to the development of high-throughput screenings, the release of drug and signatures databases and the advancements in computational biology methods, drug repositioning has become a viable business model, and AI-based drug repositioning is now the core proposition of a large number of platform companies. Moreover, the need for accelerated discovery of therapeutic candidates for COVID-19 has turned the spotlight on the value of this drug development strategy to address worldwide emergency situations. 

### 5.2. Systems Biology Approaches for Drug Repurposing for COVID-19 Treatment

At the very beginning of the COVID-19 pandemics, Zhou and coworkers developed a network medicine platform for antiviral drug repurposing [79]. The platform set the relationships between the virus–host interactome and drug targets in the human PPI network. All human proteins associated with known CoVs were collected from literature and used to generate a global host–CoVs network. Then, the proximity between proteins in the network and drug targets was calculated to select candidate repurposable drugs for human CoVs. Network-based predictions were validated by GSEA [80] and top candidates were further prioritized for drug combinations using a network-based method. Altogether, the rationale was that proteins that are functionally associated with CoVs are localized in the corresponding subnetwork within the comprehensive human PPI network, and proteins that serve as drug targets for a specific disease may also be suitable drug targets for potential antiviral infection if they are members of the same “community” [81]. Eventually, the methodology led to the identification of potentially repurposable drug classes, such as selective estrogen receptors modulators, angiotensin receptors blockers, immunosuppressant or antineoplastic agents, and anti-inflammatory agents, either alone or in combination [79]. Indeed, the number of possible drug pairs is increasing quickly; therefore, a network-based methodology is necessary to identify and validate effective combinations [82]. 

In their further paper, Zhou and coworkers described a more thorough investigation of COVID-19 biology by means of bioinformatics and network medicine [35]. They made inference on COVID-19 pathogenesis and symptoms by building a global SARS-CoV-2 virus–host interactome by merging three sources: differential transcriptomics data from primary human bronchial epithelial cells infected with SARS-CoV-2, differential proteomics data of SARS-CoV-2-infected Caco-2 cells, and the global host-CoVs PPI network described above [79]. Drug repurposing modeling over all these networks led to the identification of 34 drugs that were significantly associated with the SARS-CoV-2 datasets.

CovMulNet19 is a comprehensive COVID-19 network to be used as a network medicine tool to explore drug repurposing by taking into account several factors, from molecular interactions to symptoms, in a multivariate way [83]. The network was constructed by retrieving all available interactions involving SARS-CoV-2 proteins, human proteins interacting with them, diseases and symptoms that are related to these human proteins, and compounds that can potentially target them. The authors highlighted the over-representation of GO terms, symptoms, diseases, and drugs. Among the latter, several BCL-2 inhibitors (e.g., A-385358, obatoclax mesylate, abossipol, sabutoclax, and ABT-737) were indicated as repurposable for antiviral drug development. Additionally, two Janus kinase inhibitors (momelotinib and XL-019) were identified as top-scoring candidates.

Another case of a potential drug candidate for COVID-19 identified using symbolic AI and deep neural networks is that of baricitinib, a Janus kinase inhibitor [84]. The methodology used is based on Monte Carlo tree search and symbolic AI for the discovery of retrosynthetic routes to plan the synthesis of small organic molecules [85]. This methodology evidenced AP-2 associated kinase 1 (AAK1), cyclin g-associated kinase (GAK), and Janus kinase 1/2 (JAK) as top-scoring targets, and fedratinib, erlotinib, sunitinib, and baricitinib as top drug candidates, the latter having the highest score [84]. To date, 21 clinical trials (interventional studies) were registered for treatment of COVID-19 with baricitinib alone or as part of combination therapy (NCT04320277, NCT04321993, NCT04340232, NCT04346147, NCT04358614, NCT04373044, NCT04381936, NCT04390464, NCT04393051, NCT04399798, NCT04401579, NCT04421027, NCT04640168, NCT04693026, NCT04890626, NCT04891133, NCT04970719, NCT04832880, NCT05056558, NCT05074420, and NCT05082714). 

Table 3 summarizes all drugs that emerged from one or more network-based prediction. Several clinical trials include now one or more of these drugs. A comprehensive list of finished or ongoing interventional studies that evaluate candidates for drug repositioning can be downloaded from clinicaltrials.gov in tabular form.

More recently, Morselli Gysi and coworkers developed a network–medicine framework for drug repurposing for the treatment of COVID-19 by assembling a human interactome from 21 public databases and implementing three algorithms relying on AI, network diffusion, and network proximity to prioritize and screen FDA-approved drugs [86]. The group combined the predictions of the different pipelines in a multimodal approach in which the network communities prioritization algorithm CRank [87] offered the most reliable performance. The top-ranked drug was ritonavir, which is currently under investigation in more than 30 active interventional clinical trials to treat COVID-19. Ritonavir is being evaluated mainly in combination with other protease inhibitors, such as lopinavir (even though previous studies concluded that this combination is not significantly beneficial to severe COVID-19 patients) [88]) or PF-07321332 (also known as nirmatrelvir), a 3CL protease inhibitor developed by Pfizer. Ritonavir/PF-07321332 was recently shown to reduce the risk of hospitalization or death when administered within 3 or 5 days of symptom onset in non-hospitalized patients with mild-to-moderate COVID-19, who were at high risk of progression to severe disease. The US FDA recently issued an emergency use authorization for ritonavir/PF-07321332, marketed under the name Paxlovid™ (nirmatrelvir tablets and ritonavir tablets, co-packaged for oral use) for the treatment of mild-to-moderate COVID-19 in adult and adolescent patients who are at high risk of progression to severe COVID-19 [89]. EMA is currently evaluating Paxlovid™ for a conditional marketing authorization for the same indication [90]. Among the top 200 consensus predictions of the drug-repurposing pipelines aggregated using CRank, 13 drugs showed efficacy against SARS-CoV-2 in VeroE6 cells in a large experimental screening conducted by the same group, and 6 of them reduced viral load in Huh7 cells infected with SARS-CoV-2 (auranofin, azelastine, digoxin, vinblastine, fluvastatin, and methodextrate). 

While virtual screenings by molecular docking are limited to molecules that are supposed to directly bind a certain viral protein and/or its host target, network-based approaches allow for the identification of drugs whose mechanism of action relies on other targets (e.g., host protein targets which induce network perturbations), which is a key feature for drug development in the context of complex diseases. 

A combination of different techniques can provide constructive insight, as shown by De Siqueira Santos and coworkers [91], who exploited machine learning and network medicine as complementary approaches for drug repositioning for COVID-19. For the first purpose, the group developed a matrix decomposition algorithm to rank broad-spectrum antivirals and predict effective drug–virus relationships. The network-inspired approach was based on the interactome built by Morselli Gysi et al. and allowed to rank FDA-approved drugs with known targets using five different kernels on graphs and weighting the host proteins with differential gene expression data to understand and quantify the relevance of the perturbations induced on the COVID-19 disease module. Lastly, the group released the online tool CoREx (https://paccanarolab.org/corex; accessed on 14 January 2022), allowing users to submit a list of drugs and explore the effects on the SARS-CoV-2 host protein subnetwork by performing functional and interactome analyses. It is also possible to consider drug combinations as they may have synergistic effect.

A novel pipeline based on graph neural networks has been proposed by Hsieh and coworkers to prioritize repurposable drugs to treat COVID-19 [92]. The proposed pipeline systematically integrates the interaction between COVID-19 and drugs, deep graph neural networks, and in vitro/population-based validations. This approach allowed the authors to identify 22 top-ranked drugs, including azithromycin, atorvastatin, aspirin, and salbutamol, and potentially active drug combinations from several drug categories, with complementary exposure patterns (etoposide and sirolimus, mefloquine and sirolimus, losartan and ribavirin, and hydroxychloroquine and melatonin).

Another recent tool that can be exploited for drug discovery and development is the COVID-19 Disease Map (C19DMap), an open access repository of computational diagrams and models of molecular mechanisms assembled by over 20 independent biocuration teams [93]. 

## 6. Perspectives and Future Directions

The effort of the scientific community in understanding the pathobiology of COVID-19 and identifying novel therapeutic strategies is the response to an urgent need to face the present pandemic and reduce its impact not only on human health but also on economics and society in general terms. This urgency boosted the development of integrated omics strategies, systems biology instruments, and drug repurposing tools that look very promising, not only in the context of the COVID-19 pandemic.

A relevant example of such integrated approaches is the recent development of the 3D-SARS2 structural interactome browser by Wierbowski and coworkers [94]. To facilitate the exploration of how pathogen–host interactions might affect SARS-CoV-2 transmission and virulence, they performed interface prediction followed by molecular docking to generate a 3D structural interactome between SARS-CoV-2 and a human. This tool (http://3D-SARS2.yulab.org; accessed on 13 January 2022) represents a key resource in informing hypothesis-driven exploration of the mechanisms of SARS-CoV-2 pathology and host response. Moreover, this web server will continue to grow with the results of ongoing and future interactome studies between SARS-CoV-2 and human proteins. Given the high mutation rate of the Omicron variant and the availability of a structural model of the Omicron spike protein [95], the server could help in the investigation of variant-specific pathology. Eventually, the framework might also be rapidly deployed to analyze future viruses.

The recent emergence of the Omicron variant (see Table 1 and references therein) will for sure affect the interactome scenario, which is evolving at a fast speed and could lead to the identification of new mechanisms for host–virus interplay. On the other hand, the possibility to apply omics strategies to investigate disease pathobiology or to develop classifiers as prognostic markers or surrogate endpoints is not influenced by the presence of variants.

Overall, independently of the field of application, future directions of biomedicine are on the route of the integration of omics data at different levels by means of systems biology tools, to generate complex networks representing a disease state or a patient-specific condition. Significant information about molecular pathogenesis, prognosis, and drug response can be obtained by querying these networks with properly designed tools. Additionally, the systems biology approach may provide a useful tool for the identification of antigens to be considered for the development of new vaccines [96].

## Figures and Tables

**Table 1:** SARS-CoV-2 variants of concern (VOC).

WHO Label	First Detection	Spike Mutations of Interest ^a^	Impact on Transmissibility ^b^	Impact on Immunity ^b^	Impact on Severity ^b^
Beta	South Africa, September 2020	K417N, E484K, N501Y, D614G, A701V	Increased [8]	Increased [9,10]	Increased [11]
Gamma	Brazil, December 2020	K417T, E484K, N501Y, D614G, H655Y	Increased [12]	Increased [13]	Increased [11]
Delta	India, December 2020	L452R, T478K, D614G, P681R	Increased [14]	Increased [15]	Increased [16]
Omicron	South Africa and Botswana, November 2021	A67V, Δ69-70, T95I, G142D, Δ143-145, N211I, Δ212, ins215EPE, G339D, S371L, S373P, S375F, K417N, N440K, G446S, S477N, T478K, E484A, Q493R, G496S, Q498R, N501Y, Y505H, T547K, D614G, H655Y, N679K, P681H, N764K, D796Y, N856K, Q954H, N969K, L981F	Unclear [17,18,19]	Increased [20]	Unclear [21,22]

^a^ The list includes variations in the receptor binding domain (residues 319–541), in the S1/S2 junction and in a small stretch on S2 (residues 613–705), and any additional unusual changes specific to the variant. ^b^ “Increased” means that the property is different enough for the variant compared with previously circulating variants. “Unclear” means that the current evidence is preliminary or contradictory.

**Table 2:** Diagnostic techniques available for SARS-CoV-2 infection.

Diagnostic Strategy	Techniques	Description
Detection of viral RNA	Manual or automated nucleic acid amplification tests (NAAT): real time reverse transcription polymerase chain reaction (rRT-PCR)	Detection of structural (envelope (E), nucleocapsid (N), spike (S)) and non-structural (RNA-dependent RNA polymerase (RdRP)) protein-encoding viral genes; high sensitivity; high specificity; efficient detection of VOC; time consuming; moderate costs.
Detection of viral antigens	Immunodiagnostic techniques: lateral flow assay (LFA), commonly called rapid diagnostic tests or Ag-RDTs	Detection of viral proteins (mainly spike) through the interaction with a specific antibody; low sensitivity; high specificity; reduced efficiency in detection of VOC; rapid; low costs.
Detection of host antibodies	Serological techniques: LFA, enzyme linked immunosorbent assay (ELISA), chemiluminescent immunoassay (CLIA)	Detection of host antibodies against SARS-CoV-2; moderate sensitivity; high specificity; uncertain efficiency in detection of VOC; either rapid or time consuming, depending on the technique; low–moderate costs; useful for epidemiologic purposes; not recommended for diagnosis.

**Table 3:** Repurposable drugs prioritized by network-based prediction.

Class	Drug	Reference
Antibiotic	Azithromycin	[35]
	Tetracycline	[35]
	Cefdinir	[35]
	Cefaclor	[35]
Anti-inflammatory	Dexibuprofen	[35]
	Liftegrast	[35]
	Hydrocortisone	[35]
	Mesalazine	[79]
	Colchicine	[79]
Antimalaric	Chloroquine	[35]
	Quinacrine	[79]
Antineoplastic	Dacarbazine	[35]
	Dactinomycin	[79]
	Mercaptopurine	[79]
	Toremifene	[35,79]
Hormone	Equilin	[79]
	Megestrol acetate	[35]
	Melatonin	[35]
	Oxymetholone	[79]
JAK inhibitors	Baricitinib	[84]
	Momelotinib	[83]
	XL-019	[83]
β-Blockers	Carvedilol	[35,79]
	Timolol	[35]
	Sotalol	[35]
	Bisoprolol	[35]
	Penbutolol	[35]
β-Agonists	Procaterol	[35]
	Salbutamol	[35]
	Terbutaline	[35]
Anti-apoptotic	Obatoclax mesylate	[83]
	Abossipol	[83]
	Sabutoclax	[83]
	ABT-737	[83]
	A-385358	[83]
Angiotensin-receptor blocker	Irbesartan	[35,79]
Immunosuppressant	Sirolimus	[79]
	Temsirolimus	[35]
	Cyclosporin	[35]
	Thalidomide	[35]
	Pimecrolimus	[35]
Antiarrhythmic	Bretylium	[35]
Antidepressant	Amitriptyline	[35]
	Brexpiprazole	[35]

## Data Availability

Not applicable.

## References

[1] Wiersinga W.J., Rhodes A., Cheng A.C., Peacock S.J., Prescott H.C. (2020). Pathophysiology, Transmission, Diagnosis, and Treatment of Coronavirus Disease 2019 (COVID-19): A Review. JAMA.

[2] Latinne A., Hu B., Olival K.J., Zhu G., Zhang L., Li H., Chmura A.A., Field H.E., Zambrana-Torrelio C., Epstein J.H. (2020). Origin and cross-species transmission of bat coronaviruses in China. Nat. Commun..

[3] Zumla A., Chan J.F.W., Azhar E.I., Hui D.S.C., Yuen K.-Y. (2016). Coronaviruses—Drug discovery and therapeutic options. Nat. Rev. Drug Discov..

[4] Cheng V.C.C., Lau S.K.P., Woo P.C.Y., Yuen K.-Y. (2007). Severe Acute Respiratory Syndrome Coronavirus as an Agent of Emerging and Reemerging Infection. Clin. Microbiol. Rev..

[5] Chan J.F.-W., Lau S.K.P., To K.K.W., Cheng V.C.C., Woo P.C.Y., Yuen K.-Y. (2015). Middle East Respiratory Syndrome Coronavirus: Another Zoonotic Betacoronavirus Causing SARS-Like Disease. Clin. Microbiol. Rev..

[6] World Health Organization Pneumonia of Unknown Cause—China. https://www.who.int/csr/don/05-january-2020-pneumonia-of-unkown-cause-china/en/.

[7] Grifoni A., Sidney J., Zhang Y., Scheuermann R.H., Peters B., Sette A. (2020). A Sequence Homology and Bioinformatic Approach Can Predict Candidate Targets for Immune Responses to SARS-CoV-2. Cell Host Microbe.

[8] Tegally H., Wilkinson E., Giovanetti M., Iranzadeh A., Fonseca V., Giandhari J., Doolabh D., Pillay S., San E.J., Msomi N. (2021). Detection of a SARS-CoV-2 variant of concern in South Africa. Nature.

[9] Cele S., Africa N.F.G.S.I.S., Gazy I., Jackson L., Hwa S.-H., Tegally H., Lustig G., Giandhari J., Pillay S., Wilkinson E. (2021). Escape of SARS-CoV-2 501Y.V2 from neutralization by convalescent plasma. Nature.

[10] Madhi S.A., Baillie V., Cutland C.L., Voysey M., Koen A.L., Fairlie L., Padayachee S.D., Dheda K., Barnabas S.L., Bhorat Q.E. (2021). Efficacy of the ChAdOx1 nCoV-19 COVID-19 Vaccine against the B.1.351 Variant. N. Engl. J. Med..

[11] Funk T., Pharris A., Spiteri G., Bundle N., Melidou A., Carr M., Gonzalez G., Garcia-Leon A., Crispie F., O’Connor L. (2021). Characteristics of SARS-CoV-2 variants of concern B.1.1.7, B.1.351 or P.1: Data from seven EU/EEA countries, weeks 38/2020 to 10/2021. Eurosurveillance.

[12] Faria N.R., Mellan T.A., Whittaker C., Claro I.M., Candido D.D.S., Mishra S., Crispim M.A.E., Sales F.C.S., Hawryluk I., McCrone J.T. (2021). Genomics and epidemiology of the P.1 SARS-CoV-2 lineage in Manaus, Brazil. Science.

[13] Dejnirattisai W., Zhou D., Supasa P., Liu C., Mentzer A.J., Ginn H.M., Zhao Y., Duyvesteyn H.M., Tuekprakhon A., Nutalai R. (2021). Antibody evasion by the P.1 strain of SARS-CoV-2. Cell.

[14] Public Health England (2021). SARS-CoV-2 Variants of Concern and Variants under Investigation in England. Technical Briefing 12. https://assets.publishing.service.gov.uk/government/uploads/system/uploads/attachment_data/file/1018547/Technical_Briefing_23_21_09_16.pdf.

[15] Sheikh A., McMenamin J., Taylor B., Robertson C. (2021). SARS-CoV-2 Delta VOC in Scotland: Demographics, risk of hospital admission, and vaccine effectiveness. Lancet.

[16] Public Health England (2021). SARS-CoV-2 Variants of Concern and Variants under Investigation in England. Technical Briefing 18. https://assets.publishing.service.gov.uk/government/uploads/system/uploads/attachment_data/file/1001358/Variants_of_Concern_VOC_Technical_Briefing_18.pdf.

[17] Peacock T.P., Brown J.C., Zhou J., Thakur N., Newman J., Kugathasan R., Sukhova K., Kaforou M., Bailey D., Barclay W.S. (2022). The SARS-CoV-2 Variant, Omicron, Shows Rapid Replication in Human Primary Nasal Epithelial Cultures and Efficiently Uses the Endosomal Route of Entry. bioRxiv.

[18] Lyngse F.P., Mortensen L.H., Denwood M.J., Christiansen L.E., Møller C.H., Skov R.L., Spiess K., Fomsgaard A., Lassaunière M.M., Rasmussen M. (2021). SARS-CoV-2 Omicron VOC Transmission in Danish Households. medRxiv.

[19] UK Health Security Agency (2021). SARS-CoV-2 Variants of Concern and Variants under Investigation in England. Technical Briefing 33. https://assets.publishing.service.gov.uk/government/uploads/system/uploads/attachment_data/file/1043807/technical-briefing-33.pdf.

[20] Pulliam J.R.C., Schalkwyk C.V., Govender N., Gottberg A.V., Cohen C., Groome M.J., Dushoff J., Mlisana K., Moultrie H. (2021). Increased Risk of SARS-CoV-2 Reinfection Associated with Emergence of the Omicron Variant in South Africa. medRxiv.

[21] Wolter N., Jassat W., Walaza S., Welch R., Moultrie H., Groome M., Amoako D.G., Everatt J., Bhiman J.N., Scheepers C. (2021). Early Assessment of the Clinical Severity of the SARS-CoV-2 Omicron Variant in South Africa. medRxiv.

[22] UK Health Security Agency (2021). Omicron VOC-21NOV-01 (B.1.1.529) Technical Briefing: Hospitalisation and Vaccine Effectiveness. https://assets.publishing.service.gov.uk/government/uploads/system/uploads/attachment_data/file/1045619/Technical-Briefing-31-Dec-2021-Omicron_severity_update.pdf.

[23] Azzi L., Baj A., Alberio T., Lualdi M., Veronesi G., Carcano G., Ageno W., Gambarini C., Maffioli L., Di Saverio S. (2020). Rapid Salivary Test suitable for a mass screening program to detect SARS-CoV-2: A diagnostic accuracy study. J. Infect..

[24] Azzi L., Maurino V., Baj A., Dani M., D’Aiuto A., Fasano M., Lualdi M., Sessa F., Alberio T. (2020). Diagnostic Salivary Tests for SARS-CoV-2. J. Dent. Res..

[25] Wang Y., Teunis P. (2020). Strongly Heterogeneous Transmission of COVID-19 in Mainland China: Local and Regional Variation. Front. Med..

[26] Karaderi T., Bareke H., Kunter I., Seytanoglu A., Cagnan I., Balci D., Barin B., Hocaoglu M.B., Rahmioglu N., Asilmaz E. (2020). Host Genetics at the Intersection of Autoimmunity and COVID-19: A Potential Key for Heterogeneous COVID-19 Severity. Front. Immunol..

[27] Melenotte C., Silvin A., Goubet A.-G., Lahmar I., Dubuisson A., Zumla A., Raoult D., Merad M., Gachot B., Hénon C. (2020). Immune responses during COVID-19 infection. OncoImmunology.

[28] Dinnes J., Deeks J.J., Adriano A., Berhane S., Davenport C., Dittrich S., Emperador D., Takwoingi Y., Cunningham J., Beese S. (2020). Rapid, point-of-care antigen and molecular-based tests for diagnosis of SARS-CoV-2 infection. Cochrane Database Syst. Rev..

[29] Ji T., Liu Z., Wang G., Guo X., Khan S.A., Lai C., Chen H., Huang S., Xia S., Chen B. (2020). Detection of COVID-19: A review of the current literature and future perspectives. Biosens. Bioelectron..

[30] Mak G.C., Cheng P.K., Lau S.S., Wong K.K., Lau C.S., Lam E.T., Chan R.C.W., Tsang D.N.C. (2020). Evaluation of rapid antigen test for detection of SARS-CoV-2 virus. J. Clin. Virol..

[31] Yamayoshi S., Sakai-Tagawa Y., Koga M., Akasaka O., Nakachi I., Koh H., Maeda K., Adachi E., Saito M., Nagai H. (2020). Comparison of Rapid Antigen Tests for COVID-19. Viruses.

[32] Al-Sadeq D.W., Nasrallah G.K. (2020). The incidence of the novel coronavirus SARS-CoV-2 among asymptomatic patients: A systematic review. Int. J. Infect. Dis..

[33] Gao Z., Xu Y., Sun C., Wang X., Guo Y., Qiu S., Ma K. (2020). A systematic review of asymptomatic infections with COVID-19. J. Microbiol. Immunol. Infect..

[34] Chen G., Wu D., Guo W., Cao Y., Huang D., Wang H., Wang T., Zhang X., Chen H., Yu H. (2020). Clinical and immunological features of severe and moderate coronavirus disease 2019. J. Clin. Investig..

[35] Zhou Y., Hou Y., Shen J., Mehra R., Kallianpur A., Culver D.A., Gack M.U., Farha S., Zein J., Comhair S. (2020). A network medicine approach to investigation and population-based validation of disease manifestations and drug repurposing for COVID-19. PLoS Biol..

[36] Fasano M., Monti C., Alberio T. (2016). A systems biology-led insight into the role of the proteome in neurodegenerative diseases. Expert Rev. Proteom..

[37] Tebani A., Afonso C., Marret S., Bekri S. (2016). Omics-Based Strategies in Precision Medicine: Toward a Paradigm Shift in Inborn Errors of Metabolism Investigations. Int. J. Mol. Sci..

[38] Cannataro M., Harrison A. (2021). Bioinformatics helping to mitigate the impact of COVID-19–Editorial. Briefings Bioinform..

[39] Lualdi M., Fasano M. (2018). Statistical analysis of proteomics data: A review on feature selection. J. Proteom..

[40] Zito A., Lualdi M., Granata P., Cocciadiferro D., Novelli A., Alberio T., Casalone R., Fasano M. (2021). Gene Set Enrichment Analysis of Interaction Networks Weighted by Node Centrality. Front. Genet..

[41] Sonawane A.R., Weiss S.T., Glass K., Sharma A. (2019). Network Medicine in the Age of Biomedical Big Data. Front. Genet..

[42] Piñero J., Berenstein A., González-Pérez A., Chernomoretz A., Furlong L.I. (2016). Uncovering disease mechanisms through network biology in the era of Next Generation Sequencing. Sci. Rep..

[43] Struwe W., Emmott E., Bailey M., Sharon M., Sinz A., Corrales F.J., Thalassinos K., Braybrook J., Mills C., Barran P. (2020). The COVID-19 MS Coalition—accelerating diagnostics, prognostics, and treatment. Lancet.

[44] Grenga L., Armengaud J. (2020). Proteomics in the COVID-19 Battlefield: First Semester Check-Up. Proteomics.

[45] Mehta P., McAuley D.F., Brown M., Sanchez E., Tattersall R.S., Manson J.J. (2020). COVID-19: Consider cytokine storm syndromes and immunosuppression. Lancet.

[46] Pascolini S., Vannini A., Deleonardi G., Ciordinik M., Sensoli A., Carletti I., Veronesi L., Ricci C., Pronesti A., Mazzanti L. (2020). COVID-19 and Immunological Dysregulation: Can Autoantibodies be Useful?. Clin. Transl. Sci..

[47] Wu M., Chen Y., Xia H., Wang C., Tan C.Y., Cai X., Liu Y., Ji F., Xiong P., Liu R. (2020). Transcriptional and proteomic insights into the host response in fatal COVID-19 cases. Proc. Natl. Acad. Sci. USA.

[48] Leng L., Cao R., Ma J., Mou D., Zhu Y., Li W., Lv L., Gao D., Zhang S., Gong F. (2020). Pathological features of COVID-19-associated lung injury: A preliminary proteomics report based on clinical samples. Signal Transduct. Target. Ther..

[49] Haas P., Muralidharan M., Krogan N.J., Kaake R.M., Hüttenhain R. (2021). Proteomic Approaches to Study SARS-CoV-2 Biology and COVID-19 Pathology. J. Proteome Res..

[50] Praissman J.L., Wells L. (2021). Proteomics-Based Insights into the SARS-CoV-2–Mediated COVID-19 Pandemic: A Review of the First Year of Research. Mol. Cell. Proteom..

[51] Shu T., Ning W., Wu D., Xu J., Han Q., Huang M., Zou X., Yang Q., Yuan Y., Bie Y. (2020). Plasma Proteomics Identify Biomarkers and Pathogenesis of COVID-19. Immunity.

[52] Monti C., Zilocchi M., Colugnat I., Alberio T. (2018). Proteomics turns functional. J. Proteom..

[53] Perfetto L., Pastrello C., Del-Toro N., Duesbury M., Iannuccelli M., Kotlyar M., Licata L., Meldal B., Panneerselvam K., Panni S. (2020). The IMEx coronavirus interactome: An evolving map of Coronaviridae–host molecular interactions. Database.

[54] Iacobucci I., Monaco V., Cozzolino F., Monti M. (2020). From classical to new generation approaches: An excursus of -omics methods for investigation of protein-protein interaction networks. J. Proteom..

[55] Feng S., Zhou L., Huang C., Xie K., Nice E.C. (2014). Interactomics: Toward protein function and regulation. Expert Rev. Proteom..

[56] Imai Y., Kuba K., Rao S., Huan Y., Guo F., Guan B., Yang P., Sarao R., Wada T., Leong-Poi H. (2005). Angiotensin-converting enzyme 2 protects from severe acute lung failure. Nature.

[57] Gheblawi M., Wang K., Viveiros A., Nguyen Q., Zhong J.-C., Turner A.J., Raizada M.K., Grant M.B., Oudit G.Y. (2020). Angiotensin-Converting Enzyme 2: SARS-CoV-2 Receptor and Regulator of the Renin-Angiotensin System: Celebrating the 20th Anniversary of the Discovery of ACE2. Circ. Res..

[58] Lite C., Ahmed S.S.S.J., Juliet M., Freddy A.J. (2021). SARS-CoV-2/human interactome reveals ACE2 locus crosstalk with the immune regulatory network in the host. Pathog. Dis..

[59] Bamberger C., Pankow S., Martínez-Bartolomé S., Diedrich J., Park R., Yates J. (2021). The Host Interactome of Spike Expands the Tropism of SARS-CoV-2. bioRxiv.

[60] Gordon D.E., Jang G., Bouhaddou M., Krogan N.J. (2020). A SARS-CoV-2 protein interaction map reveals targets for drug repurposing. Nature.

[61] Perrin-Cocon L., Diaz O., Jacquemin C., Barthel V., Ogire E., Ramière C., André P., Lotteau V., Vidalain P.-O. (2020). The current landscape of coronavirus-host protein–protein interactions. J. Transl. Med..

[62] Corsello S., Bittker J.A., Liu Z., Gould J., McCarren P., Hirschman J.E., Johnston S.E., Vrcic A., Wong B., Khan M. (2017). The Drug Repurposing Hub: A next-generation drug library and information resource. Nat. Med..

[63] Messina F., Giombini E., Agrati C., Vairo F., Bartoli T.A., Al Moghazi S., Piacentini M., Locatelli F., Kobinger G., Maeurer M. (2020). COVID-19: Viral–host interactome analyzed by network based-approach model to study pathogenesis of SARS-CoV-2 infection. J. Transl. Med..

[64] Shen B., Yi X., Sun Y., Bi X., Du J., Zhang C., Quan S., Zhang F., Sun R., Qian L. (2020). Proteomic and Metabolomic Characterization of COVID-19 Patient Sera. Cell.

[65] Su Y., Chen D., Yuan D., Lausted C., Choi J., Dai C.L., Voillet V., Duvvuri V.R., Scherler K., Troisch P. (2020). Multi-Omics Resolves a Sharp Disease-State Shift between Mild and Moderate COVID-19. Cell.

[66] Messner C.B., Demichev V., Wendisch D., Michalick L., White M., Freiwald A., Textoris-Taube K., Vernardis S.I., Egger A.-S., Kreidl M. (2020). Ultra-High-Throughput Clinical Proteomics Reveals Classifiers of COVID-19 Infection. Cell Syst..

[67] Chen Y., Zheng Y., Yu Y., Wang Y., Huang Q., Qian F., Sun L., Song Z., Chen Z., Feng J. (2020). Blood molecular markers associated with COVID-19 immunopathology and multi-organ damage. EMBO J..

[68] Tilocca B., Britti D., Urbani A., Roncada P. (2020). Computational Immune Proteomics Approach to Target COVID-19. J. Proteome Res..

[69] Hou X., Zhang X., Wu X., Lu M., Wang D., Xu M., Wang H., Liang T., Dai J., Duan H. (2020). Serum Protein Profiling Reveals a Landscape of Inflammation and Immune Signaling in Early-stage COVID-19 Infection. Mol. Cell. Proteom..

[70] Xiao F., Tang M., Zheng X., Liu Y., Li X., Shan H. (2020). Evidence for Gastrointestinal Infection of SARS-CoV-2. Gastroenterology.

[71] Young B.E., Ong S.W.X., Kalimuddin S., Low J.G., Tan S.Y., Loh J., Ng O.T., Marimuthu K., Ang L.W., Mak T.M. (2020). Epidemiologic Features and Clinical Course of Patients Infected With SARS-CoV-2 in Singapore. JAMA.

[72] Hoehl S., Rabenau H., Berger A., Kortenbusch M., Cinatl J., Bojkova D., Behrens P., Böddinghaus B., Götsch U., Naujoks F. (2020). Evidence of SARS-CoV-2 Infection in Returning Travelers from Wuhan, China. N. Engl. J. Med..

[73] Bojkova D., Klann K., Koch B., Widera M., Krause D., Ciesek S., Cinatl J., Münch C. (2020). Proteomics of SARS-CoV-2-infected host cells reveals therapy targets. Nature.

[74] Sahoo D., Katkar G.D., Khandelwal S., Behroozikhah M., Claire A., Castillo V., Tindle C., Fuller M., Taheri S., Rogers T.F. (2021). AI-guided discovery of the invariant host response to viral pandemics. EBioMedicine.

[75] Liu X., Huuskonen S., Laitinen T., Redchuk T., Bogacheva M., Salokas K., Pöhner I., Öhman T., Tonduru A.K., Hassinen A. (2021). SARS-CoV-2–host proteome interactions for antiviral drug discovery. Mol. Syst. Biol..

[76] Nisar T., Sutherland-Foggio H., Husar W. (2019). Antiviral amantadine. Lancet Neurol..

[77] Hubsher G., Haider M., Okun M. (2012). Amantadine: The journey from fighting flu to treating Parkinson disease. Neurology.

[78] Thomas D., Wessel C. (2015). BIO Report–Venture Funding for Therapeutic Innovation (2015).

[79] Zhou Y., Hou Y., Shen J., Huang Y., Martin W., Cheng F. (2020). Network-based drug repurposing for novel coronavirus 2019-nCoV/SARS-CoV-2. Cell Discov..

[80] Subramanian A., Tamayo P., Mootha V.K., Mukherjee S., Ebert B.L., Gillette M.A., Paulovich A., Pomeroy S.L., Golub T.R., Lander E.S. (2005). Gene set enrichment analysis: A knowledge-based approach for interpreting genome-wide expression profiles. Proc. Natl. Acad. Sci. USA.

[81] Su G., Kuchinsky A., Morris J., States D.J., Meng F. (2010). GLay: Community structure analysis of biological networks. Bioinformatics.

[82] Zhou Y., Wang F., Tang J., Nussinov R., Cheng F. (2020). Artificial intelligence in COVID-19 drug repurposing. Lancet Digit. Heal..

[83] Verstraete N., Jurman G., Bertagnolli G., Ghavasieh A., Pancaldi V., De Domenico M. (2020). CovMulNet19, Integrating Proteins, Diseases, Drugs, and Symptoms: A Network Medicine Approach to COVID-19. Netw. Syst. Med..

[84] Richardson P., Griffin I., Tucker C., Smith D., Oechsle O., Phelan A., Rawling M., Savory E., Stebbing J. (2020). Baricitinib as potential treatment for 2019-nCoV acute respiratory disease. Lancet.

[85] Segler M.H.S., Preuss M., Waller M.P. (2018). Planning chemical syntheses with deep neural networks and symbolic AI. Nature.

[86] Gysi D.M., Do Valle Í., Zitnik M., Ameli A., Gan X., Varol O., Ghiassian S.D., Patten J.J., Davey R.A., Loscalzo J. (2021). Network medicine framework for identifying drug-repurposing opportunities for COVID-19. Proc. Natl. Acad. Sci. USA.

[87] Zitnik M., Sosič R., Leskovec J. (2018). Prioritizing network communities. Nat. Commun..

[88] Cao B., Wang Y., Wen D., Liu W., Wang J., Fan G., Ruan L., Song B., Cai Y., Wei M. (2020). A Trial of Lopinavir–Ritonavir in Adults Hospitalized with Severe COVID-19. N. Engl. J. Med..

[89] US Food and Drug Administration Coronavirus (COVID-19) Update: FDA Authorizes First Oral Antiviral for Treatment of COVID-19. https://www.fda.gov/news-events/press-announcements/coronavirus-covid-19-update-fda-authorizes-first-oral-antiviral-treatment-covid-19.

[90] European Medicines Agency EMA Receives Application for Conditional Marketing Authorisation Paxlovid (PF-07321332 and Ritonavir) Treating Patients with COVID-19. https://www.ema.europa.eu/en/news/ema-receives-application-conditional-marketing-authorisation-paxlovid-pf-07321332-ritonavir-treating.

[91] Santos S.D.S., Torres M., Galeano D., Sánchez M.D.M., Cernuzzi L., Paccanaro A. (2022). Machine learning and network medicine approaches for drug repositioning for COVID-19. Patterns.

[92] Hsieh K., Wang Y., Chen L., Zhao Z., Savitz S., Jiang X., Tang J., Kim Y. (2021). Drug repurposing for COVID-19 using graph neural network and harmonizing multiple evidence. Sci. Rep..

[93] Ostaszewski M., Niarakis A., Mazein A., Kuperstein I., Phair R., Orta-Resendiz A., Singh V., Aghamiri S.S., Acencio M.L., Glaab E. (2021). COVID19 Disease Map, a computational knowledge repository of virus–host interaction mechanisms. Mol. Syst. Biol..

[94] Wierbowski S.D., Liang S., Liu Y., Chen Y., Gupta S., Andre N.M., Lipkin S.M., Whittaker G.R., Yu H. (2021). A 3D structural SARS-CoV-2–human interactome to explore genetic and drug perturbations. Nat. Methods.

[95] Fantini J., Yahi N., Colson P., Chahinian H., La Scola B., Raoult D. (2022). The puzzling mutational landscape of the SARS-2-variant Omicron. J. Med Virol..

[96] McConnell M.J., Martín-Galiano A.J. (2021). Designing Multi-Antigen Vaccines Against Acinetobacter baumannii Using Systemic Approaches. Front. Immunol..

